# Vitamin K-Antagonists Accelerate Atherosclerotic Calcification and Induce a Vulnerable Plaque Phenotype

**DOI:** 10.1371/journal.pone.0043229

**Published:** 2012-08-29

**Authors:** Leon J. Schurgers, Ivo A. Joosen, Eduard M. Laufer, Martijn L. L. Chatrou, Marjolein Herfs, Mark H. M. Winkens, Ralf Westenfeld, Verena Veulemans, Thilo Krueger, Catherine M. Shanahan, Willi Jahnen-Dechent, Erik Biessen, Jagat Narula, Cees Vermeer, Leonard Hofstra, Chris P. Reutelingsperger

**Affiliations:** 1 Department of Biochemistry, Cardiovascular Research Institute Maastricht, Maastricht University Medical Centre, Maastricht, The Netherlands; 2 Department of Cardiology, Cardiovascular Research Institute Maastricht, Maastricht University Medical Centre, Maastricht, The Netherlands; 3 VitaK, Maastricht University, Maastricht, The Netherlands; 4 Department of Cardiology, Cardiovascular Research Institute Maastricht, Maastricht University Medical Centre, Maastricht, The Netherlands; 5 Division of Cardiology, University Hospital Düsseldorf, Düsseldorf, Germany; 6 Division of Nephrology and Clinical Immunology, RWTH Aachen University, Aachen, Germany; 7 Cardiovascular Division, King's College London, London, United Kingdom; 8 Department of Biomedical Engineering, RWTH Aachen University, Aachen, Germany; 9 Department of Pathology, Maastricht University Medical Centre, Maastricht, The Netherlands; 10 Department of Cardiology, Mount Sinai School of Medicine, New York, New York, United States of America; 11 Cardiology Center Netherlands, Utrecht, The Netherlands; Brigham and Women's Hospital, Harvard Medical School, United States of America

## Abstract

**Background:**

Vitamin K-antagonists (VKA) are treatment of choice and standard care for patients with venous thrombosis and thromboembolic risk. In experimental animal models as well as humans, VKA have been shown to promote medial elastocalcinosis. As vascular calcification is considered an independent risk factor for plaque instability, we here investigated the effect of VKA on coronary calcification in patients and on calcification of atherosclerotic plaques in the ApoE^−/−^ model of atherosclerosis.

**Methodology/Principal Findings:**

A total of 266 patients (133 VKA users and 133 gender and Framingham Risk Score matched non-VKA users) underwent 64-slice MDCT to assess the degree of coronary artery disease (CAD). VKA-users developed significantly more calcified coronary plaques as compared to non-VKA users. ApoE^−/−^ mice (10 weeks) received a Western type diet (WTD) for 12 weeks, after which mice were fed a WTD supplemented with vitamin K_1_ (VK_1_, 1.5 mg/g) or vitamin K_1_ and warfarin (VK_1_&W; 1.5 mg/g & 3.0 mg/g) for 1 or 4 weeks, after which mice were sacrificed. Warfarin significantly increased frequency and extent of vascular calcification. Also, plaque calcification comprised microcalcification of the intimal layer. Furthermore, warfarin treatment decreased plaque expression of calcification regulatory protein carboxylated matrix Gla-protein, increased apoptosis and, surprisingly outward plaque remodeling, without affecting overall plaque burden.

**Conclusions/Significance:**

VKA use is associated with coronary artery plaque calcification in patients with suspected CAD and causes changes in plaque morphology with features of plaque vulnerability in ApoE^−/−^ mice. Our findings underscore the need for alternative anticoagulants that do not interfere with the vitamin K cycle.

## Introduction

Vitamin K antagonists (VKA) are the most frequently prescribed drugs to control blood coagulation of patients with thrombosis and patients at risk of thromboembolic events. VKA block the vitamin K epoxide reductase complex that drives conversion of certain glutamate residues of vitamin K-dependent coagulation factors into γ-carboxyglutamic acid (Gla)-residues [1]. VKA therapy may have undesired side-effects in addition to risk of bleeding because a number of proteins outside the coagulation system also require γ-glutamylcarboxylation to become biologically active [2].

Matrix Gla-protein (MGP) is a vitamin K-dependent protein not related to blood coagulation but also affected by VKA [3]. Animal models showed that MGP is a strong inhibitor of calcification of arterial vessel wall and cartilage [4]. In arteries, MGP acts as a local inhibitor of media calcification [5], [6]. Its inhibitory mechanism is still not fully understood but involves inhibition of bone morphogenetic protein 2 and 4 (BMP-2 and -4) [7], [8], suppression of osteochondrogenic transdifferentiation of vascular smooth muscle cells [9] and direct inhibition of calcium-crystal growth [10], [11]; in all cases MGP requires vitamin K-dependent γ-carboxylation [10]. Concordantly, clinical studies and case reports revealed that VKA treatment is associated with arterial calcification and upregulation of uncarboxylated MGP (ucMGP) [12], [13], [14], [15].

MGP expression is increased in human atherosclerotic lesions [16] and vascular smooth muscle cells (VSMCs) are predominantly involved in intimal calcification [17]. Overexpression of MGP in the apoE^−/−^ mouse model of atherosclerosis reduced both intimal and medial calcification of atherosclerotic plaques whereas gene deletion of MGP in apoE^−/−^ mice accelerated intimal calcification of plaques [18]. BMP-2 transgenic apoE^−/−^ mice displayed increased calcification of intima of atheromatous lesions, suggesting a key role for MGP in suppressing BMP-2 induced vascular calcification [19]. Since intimal calcification of atherosclerotic plaques is considered a risk factor for plaque rupture [20], [21] we were interested in effects of VKA on atherosclerotic intima calcification.

In this paper we report results of our study that investigated the effects of VKA on calcification of coronary atherosclerotic lesions in patients with suspected CAD using 64-slice multi detector-row computed tomography (MDCT). MDCT allows quantifying calcification of vascular tissue but is insufficient to distinguish between medial and intimal calcification. Therefore, we investigated effects of VKA on calcification of atherosclerotic plaque of apoE^−/−^ mice.

## Results

### Coronary Calcification in Patients

133 VKA users and 133 individually age, gender and FRS matched non-VKA users were included in this study. Of the 133 VKA users, 52 patients had no plaque and of the 133 non-VKA 41 patients had no plaque at time of screening. VKA users were divided in tertiles based on duration of VKA use. The mean duration of VKA use is 2.5±1.5 months in the first tertile (T1), 18.7±8.8 months in the second tertile (T2) and 86.4±47.1 months in the third tertile (T3). The categorization of the VKA users into tertiles distributed the non-VKA users also in three groups because each non-VKA user was individually matched with a VKA user. Tables 1 and 2 summarize the baseline characteristics of the tertiles of non-VKA users and VKA users, respectively.

**Table 1:** Baseline characteristics of patients on VKA treatment.

Variable	1^st^ tertile(n = 44)	2^nd^ tertile(n = 44)	3^rd^ tertile(n = 45)	*p* value
Male gender	29 (65.9)	27 (61.4)	35 (77.8)	.231
Age (years)	57.6±10.8	59.7±10.1	63.6±8.5	.016
Smoking	7 (15.9)	7 (15.9)	3 (6.7)	.325
Diabetes mellitus	3 (6.8)	1 (2.3)	3 (6.7)	.560
Positive family history	10 (22.7)	11 (25.0)	10 (22.2)	.948
Systolic BP (mmHg)	140±16	142±21	140±18	.861
Total cholesterol (mmol/L)	5.5±1.1	5.3±1.2	5.2±1.1	.294
LDL-cholesterol (mmol/L)	3.5±1.0	3.3±1.2	3.3±1.0	.537
HDL-cholesterol (mmol/L)	1.3±0.5	1.3±0.5	1.2±0.4	.349
Triglycerides (mmol/L)	1.7±1.3	1.7±1.0	1.8±1.3	.981
Framingham Risk Score	21.6±15.6	21.4±13.9	29.8±17.2	.017
Coronary calcium score (Agatston)	79.6±159.8	142.4±306.0	252.5±399.3	.029

Continuous variables are presented as means ± SD, while categorical variables are expressed as number (percentage). BP, blood pressure; HDL, high-density lipoprotein; LDL, low-density lipoprotein.

**Table 2:** Baseline characteristics of patients not on VKA treatment.

Variable	1^st^ tertile (n = 44)	2^nd^ tertile (n = 44)	3^rd^ tertile (n = 45)	*p* value
Male gender	29 (65.9)	27 (61.4)	35 (77.8)	.227
Age (years)	57.6±8.9	59.8±9.4	59.2±9.5	.536
Smoking	11 (25.0)	7 (15.9)	9 (20.0)	.569
Diabetes mellitus	5 (11.4)	1 (2.3)	4 (8.9)	.247
Positive family history	23 (52.3)	17 (38.6)	16 (35.6)	.238
Systolic BP (mmHg)	140±17	146±19	145±20	.354
Total cholesterol (mmol/L)	5.3±1.1	5.2±1.1	5.5±1.4	.581
LDL-cholesterol (mmol/L)	3.3±1.0	3.3±1.0	3.5±1.3	.470
HDL-cholesterol (mmol/L)	1.3±0.4	1.3±0.4	1.1±0.3	.111
Triglycerides (mmol/L)	1.7±0.8	1.5±0.8	2.1±1.3	.022
Framingham Risk Score	21.6±15.6	22.2±14.1	28.7±17.2	.065
Coronary calcium score (Agatston)	153.4±261.4	156.0±285.1	167.8±263.0	.965

Continuous variables are presented as means ± SD, while categorical variables are expressed as number (percentage). BP, blood pressure; HDL, high-density lipoprotein; LDL, low-density lipoprotein.

**Figure 1:**
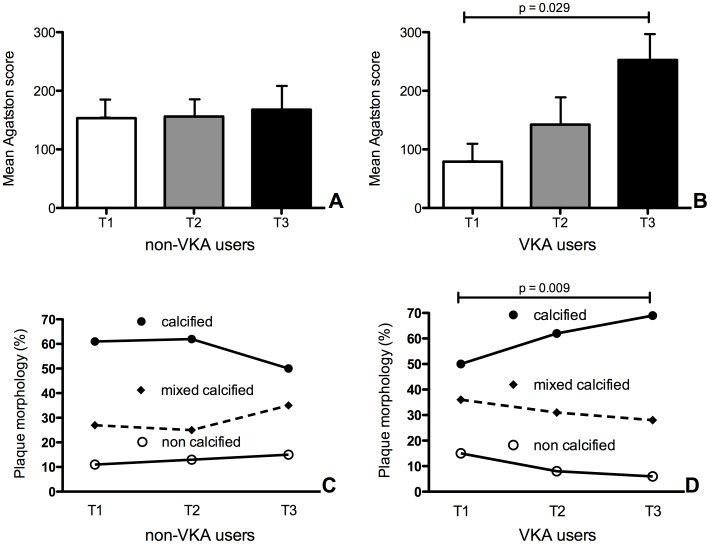
Duration of VKA treatment significantly increases the amount of coronary calcification and number of calcified stenotic plaques. A non-enhanced scan was performed to measure the Coronary artery calcification (Agatston score). Panel A shows a significant increase in Agatston score in VKA users (p = 0.029), as patients use VKA for a longer time (T = tertile of VKA use). This increase is not visible in individually matched patients not on VKA (panel B; p = 0.965). Next, CT-angiography was performed. All coronary segments were assessed for plaque presence and plaque morphology. In patients using VKA, a significant trend (p = 0.009) was seen towards a higher percentage of calcified plaques in those patients treated longest with VKA (panel C). In contrast, this was not the case for the non-VKA users (panel D). Coronary plaques were identified and categorized as calcified (closed circles), mixed calcified (diamonds) and non-calcified (open circles). T1, T2 and T3 indicate 1^st^, 2^nd^ and 3^rd^ tertile respectively.

Coronary calcification was quantified as the Agatston score. There were no significant differences between the mean Agatston score in the tertiles of non-VKA users (p = 0.965; Figure 1A). On the other hand, the mean Agatston score increased significantly in VKA users, as the duration of VKA use increased (p = 0.029; Figure 1B).

Next, we analyzed all coronary segments in each patient to assess plaque morphology and the degree of luminal stenosis. Plaques were categorized as calcified, mixed or non-calcified plaque. Plaque morphology did not differ significantly between the three tertiles of non-VKA users (Figure 1C). In contrast, the fraction of calcified coronary plaques increased significantly with prolonged VKA use. Fifty percent of the plaques in the 1^st^ tertile of VKA users were calcified, compared to 61.5% in the 2^nd^ tertile and 68.5% in the 3^rd^ tertile (p<0.01; Figure 1D). Follow-up information was available for all patients (mean follow-up time 2.9±0.6 years). In the group of VKA users, 2 patients underwent coronary revascularization, 1 patient suffered an acute coronary syndrome and 2 patients died as a result of coronary artery disease. In the group of non-VKA users, 5 patients underwent coronary revascularization, no patients suffered an acute coronary syndrome and 1 patient died as a result of coronary artery disease.

We also checked other medication use between VKA and non-VKA patients. VKA users were using Verapamil significantly more often compared to non VKA users (P<0.005). This suggests that the difference in Agatston score between the VKA users and non VKA users may be even larger, since Verapamil is an inhibitor of calcification [22]. There were no significant differences in other frequent used drugs such as beta blockers, aspirin and statins between VKA and non VKA users.

In conclusion, these results indicate that VKA use is associated with an increase of atherosclerotic plaque calcification. Size and location of calcium deposits are important determinants for plaque stability. Unfortunately, due to the limited spatial resolution of MDCT and the presence of blooming artifacts, it is not possible to assess if these calcium deposits are localized in the intima or the media of the arterial wall. Therefore we performed a more detailed study of effect of VKA on plaque calcification in an established model of atherosclerosis, the apoE^−/−^ mice.

### Effect of Warfarin on Atherosclerotic Burden of apoE^−/−^ Mice

40 apoE^−/−^ mice entered the experimental scheme at the age of 10 weeks. Vitamin K_1_ (VK_1_) and warfarin (most prescribed VKA) supplementation to the Western type diet (WTD) did neither change plasma cholesterol, calcium and phosphate levels nor body weight as compared with WTD supplemented with VK_1_ only demonstrating that warfarin was well tolerated (Table 3). Vitamin K1 was co-administered because warfarin alone would cause internal bleeding of mice. Vitamin K1 counteracts warfarin’s antagonistic activity in liver but not in extrahepatic tissue [23].

**Table 3:** Weight and blood characteristics of apoE^−/−^ mice at baseline, and after one or four weeks of treatment.

	Baseline	Control		Warfarin	
	3 month WTD	1 week	4 weeks	1 week	4 weeks
Cholesterol	11.0±0.3	11.0±0.4	10.9±0.5	11.1±0.5	11.0±0.3
Calcium	2.29±0.04	2.28±0.05	2.29±0.03	2.27±0.03	2.27±0.03
Phosphate	1.94±0.04	1.93±0.04	1.95±0.04	1.94±0.03	1.94±0.02
Weight male	21.7±0.6	21.3±1.2	23.7±0.6	22.3±0.6	23.0±1.0
Weight female	31.7±0.6	31.7±1.5	32.3±2.3	29.7±1.2	31.3±2.3

WTD, Western type diet.

**Figure 2:**
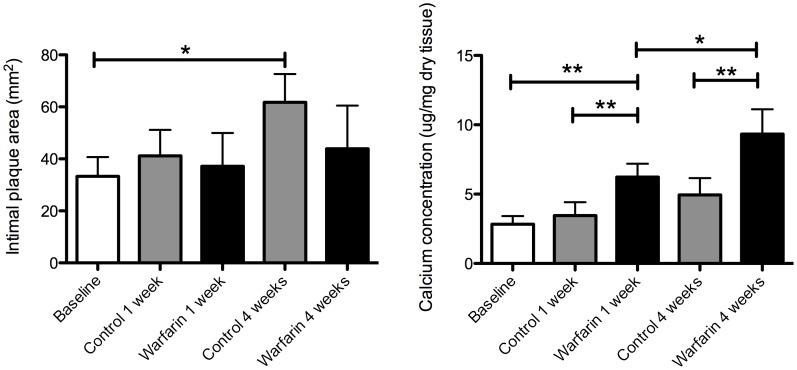
Warfarin treatment of apoE deficient mice on Western Type Diet does not influence plaque size but increases vascular calcification. ApoE^−/−^ mice developed atherosclerotic lesions in the aortic arch and carotid arteries when maintained on Western type diet (WTD) for 12 weeks (A). From baseline mice were fed with WTD plus vitamin K (VK_1_) or WTD plus vitamin K and warfarin (VK_1_&W) for the duration of one week or four weeks. Growth of intimal area was not significantly affected by warfarin. Vascular calcium was determined by AAS and revealed significant increase in calcium at 1 and 4 weeks warfarin treatment. Statistically significant differences were determined by the Kruskal Wallis test. *P<0.05.

We quantified intimal plaque area in the aortic arch by histomorphometry of hematoxylin/eosin (HE) and Masson’s trichrome stained sections. Both H/E and Masson’s trichrome staining showed that dietary supplementation of warfarin did not change plaque expansion during the 4 weeks treatment regimen (Figure 2A) and neither number nor size distribution of plaques were affected. Additionally, collagen content of the plaques did not differ significantly.

### Effect of Warfarin on Plaque Calcification

**Figure 3:**
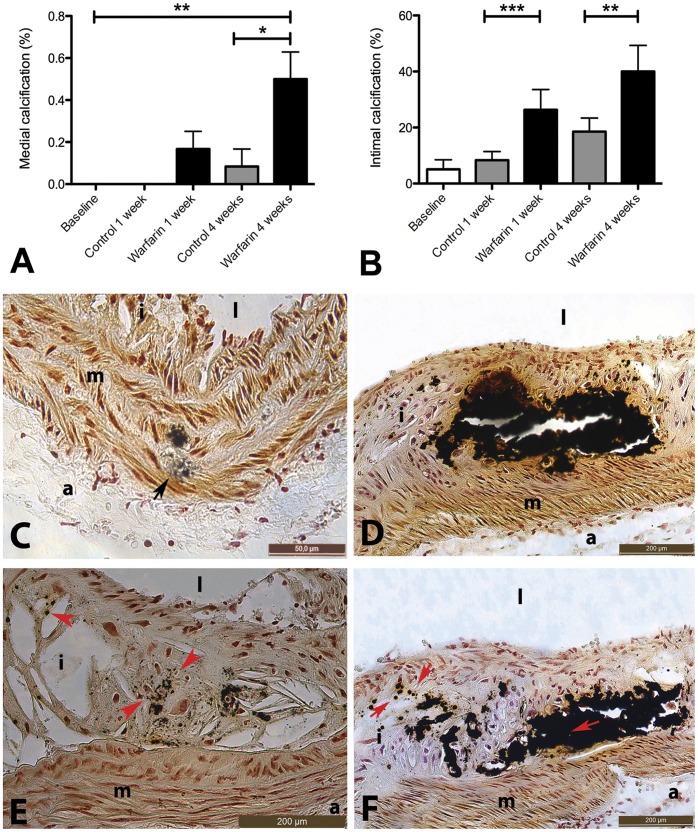
Warfarin treatment rapidly increases medial and intimal plaque calcification in apoE*^−/−^* mice. ApoE−/− mice received WTD for three month (baseline) and subsequently control diet (WTD plus VK_1_) or warfarin (WTD plus VK_1_&W). Von Kossa stained calcified plaques were scored for medial (A,C) and intimal plaque calcification (B,D). In addition calcification was categorized as microcalcification (E, arrow heads) and macrocalcification (F, arrows). Microcalcifications occur either alone or in conjunction with macrocalcification. Statistically significant differences were determined by the Kruskal Wallis test. *P<0.05, **P<0.01, ***P<0.001. i, intima; m, media; l, lumen; a, adventitia.

We quantified vascular calcification of the thoracic aorta using two different techniques. Atomic absorption spectrometry (AAS; Department of Clinical Chemistry, University Hospital Maastricht, The Netherlands) revealed that calcium levels were already elevated at one week post-baseline and increased further 4 weeks post-baseline if warfarin was added to the WTD (Figure 2B). Whereas AAS measures overall calcium of tissue, von Kossa staining allows pinpointing the actual location and morphology of intra-plaque calcium deposits. Von Kossa analysis of serial sections showed that calcium deposits were mainly confined to intimal layers of atherosclerotic lesions. No medial calcification was observed at baseline but warfarin induced significant calcification of the medial layer of the plaque (Figure 3A,C). In all cases intimal calcification was more prevalent than medial calcification (Figure 3B,D). Warfarin treatment significantly increased number of plaques with intimal calcification and also increased calcification nodule area (Figure 3B). We were also able to discriminate between macrocalcification (>50 μm) and microcalcifications (<2 μm) (Figure 3E,F). Macrocalcification was often accompanied by microcalcification (Figure 3F). Microcalcifications were also observed in absence of macrocalcification (Figure 3E). We conclude that warfarin accelerates both medial and intimal calcification of atherosclerotic plaque.

### Effect of Warfarin on Plaque Phenotype

**Figure 4:**
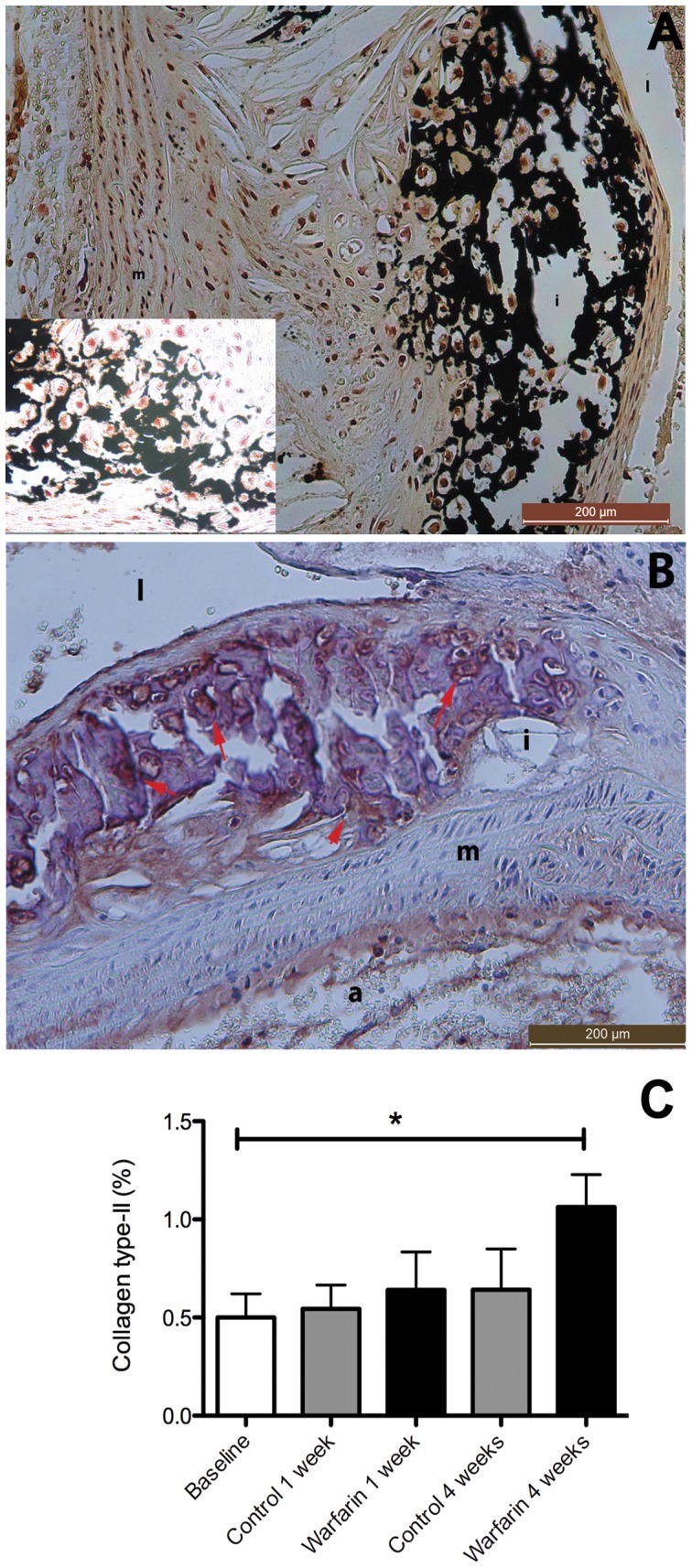
Warfarin induces atherosclerotic calcification which induces a chondrogenic plaque phenotype. To characterize cells calcified or adjacent to the calcification area we closely examined stained von Kossa sections (A). Calcified cells in the atherosclerotic plaque displayed chondrocyte features. To confirm the presence of chondrocytes we stained for collagen type-II, a specific marker for chondrocytes. Areas in the plaque with suspected chondrocytes stained positive for collagen type-II (B). Quantification revealed that after 4 weeks of warfarin treatment a significant increase was measured (C). Statistically significant differences were determined by the Kruskal Wallis test. *P<0.05. i, intima; m, media; l, lumen; a, adventitia.

**Figure 5:**
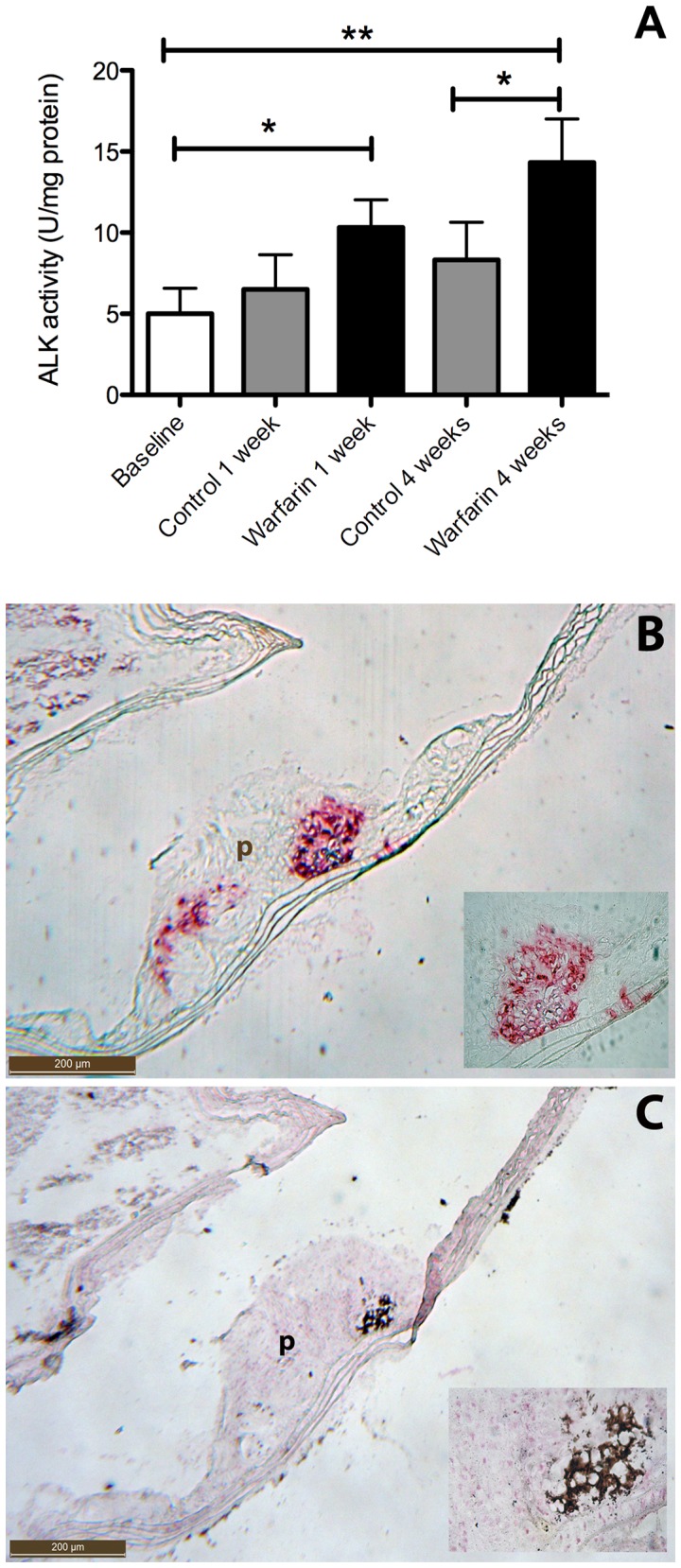
Warfarin upregulates alkaline phosphatase activity in atherosclerotic plaque. To further characterize the calcified lesions we measured (A) and stained for alkaline phosphatase (C). Areas positive for von Kossa (B) often co-stained for ALK (C). Therefore we analyzed the ALK content of the different treatment groups (A). After one week of warfarin treatment a significant increase in ALK was already noticed, which further increased after 4 weeks of warfarin. ALK expression indicates an osteo/chondrogenic differentiation of VSMCs. Statistically significant differences were determined by the Kruskal Wallis test. *P<0.05, **P<0.01. p, plaque.

Histochemistry of calcified plaques in the 4 weeks warfarin treated animals revealed abundant presence of chondrocyte like cells in close proximity to macro calcium deposits (Figure 4A). The presence of bone-associated proteins in these plaques was confirmed by collagen type-II staining (Figure 4B, C) as well as alkaline phosphatase measurement (Figure 5A) and staining (Figure 5B,C). Of note, warfarin treatment (4 weeks) did not only augment calcification but also significantly increased the presence of both chondrocytic markers.

**Figure 6:**
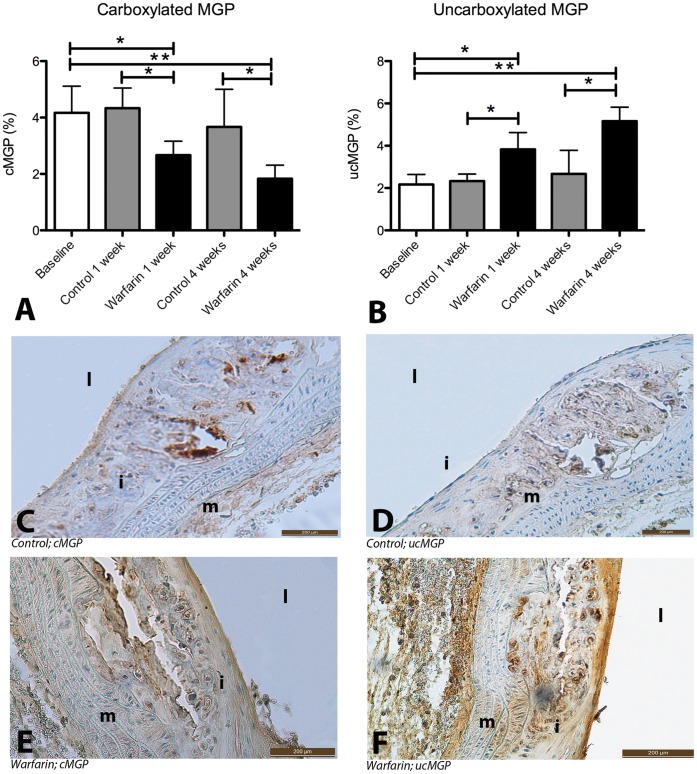
Warfarin affects carboxylation of MGP in atherosclerotic plaque. To confirm action of warfarin locally in the plaque we stained plaques for ucMGP and cMGP. UcMGP is the result of vitamin K-deficiency. Within one week of warfarin treatment the amount of cMGP (A,E) decreased significantly and decreased even further after 4 weeks of warfarin treatment. The decrease in cMGP was accompanied by an increase in ucMGP (B,F). Immunohistochemistry for cMGP and ucMGP (E,F) showed increased amounts of ucMGP compared with cMGP in apoE^−/−^ mice on warfarin, whereas apoE^−/−^ mice on control diet had predominantly cMGP (C,D). cMGP indicates carboxylated MGP; ucMGP, uncarboxylated MGP. Statistically significant differences were determined by the Kruskal Wallis test. *P<0.05, **P<0.01. i, intima; m, media; l, lumen.

As carboxylated MGP was previously shown to inhibit chondrogenesis via inactivation of BMP2 [7], we stained aortic plaques for the presence of carboxylated versus uncarboxylated MGP. Warfarin treatment caused a dramatic decrease in carboxylated MGP with a concomitant increase in uncarboxylated MGP (Figure 6A-F). mRNA expression levels of MGP were not affected by warfarin (Table 4). These results suggest that warfarin interferes with calcification phenotype in the plaque by inhibiting carboxylation of MGP. We also measured mRNA expression levels of MMP-2 and MMP-9 which were also not significantly different by 4-weeks warfarin administration compared to baseline (Table 4).

**Table 4:** mRNA expression levels of aortic tissue (n = 3) of calcification biomarkers at control and warfarin treatment at 4 weeks time point.

Marker	Control		Warfarin & K1		Significance
	mean	SD	mean	SD	P =
SM22a	1.4	0.7	1.4	0.5	0.783
Runx2	2.0	0.7	1.9	1.1	0.946
BMP-2	1.3	0.6	1.4	0.3	0.967
BMP-4	2.2	1.6	2.2	0.9	0.825
MGP	1.3	0.7	1.9	0.4	0.292
VEGF	1.4	0.6	1.3	0.3	0.938
MMP-2	1.2	0.5	1.4	0.5	0.891
MMP-9	1.3	0.6	1.9	0.5	0.204

**Figure 7:**
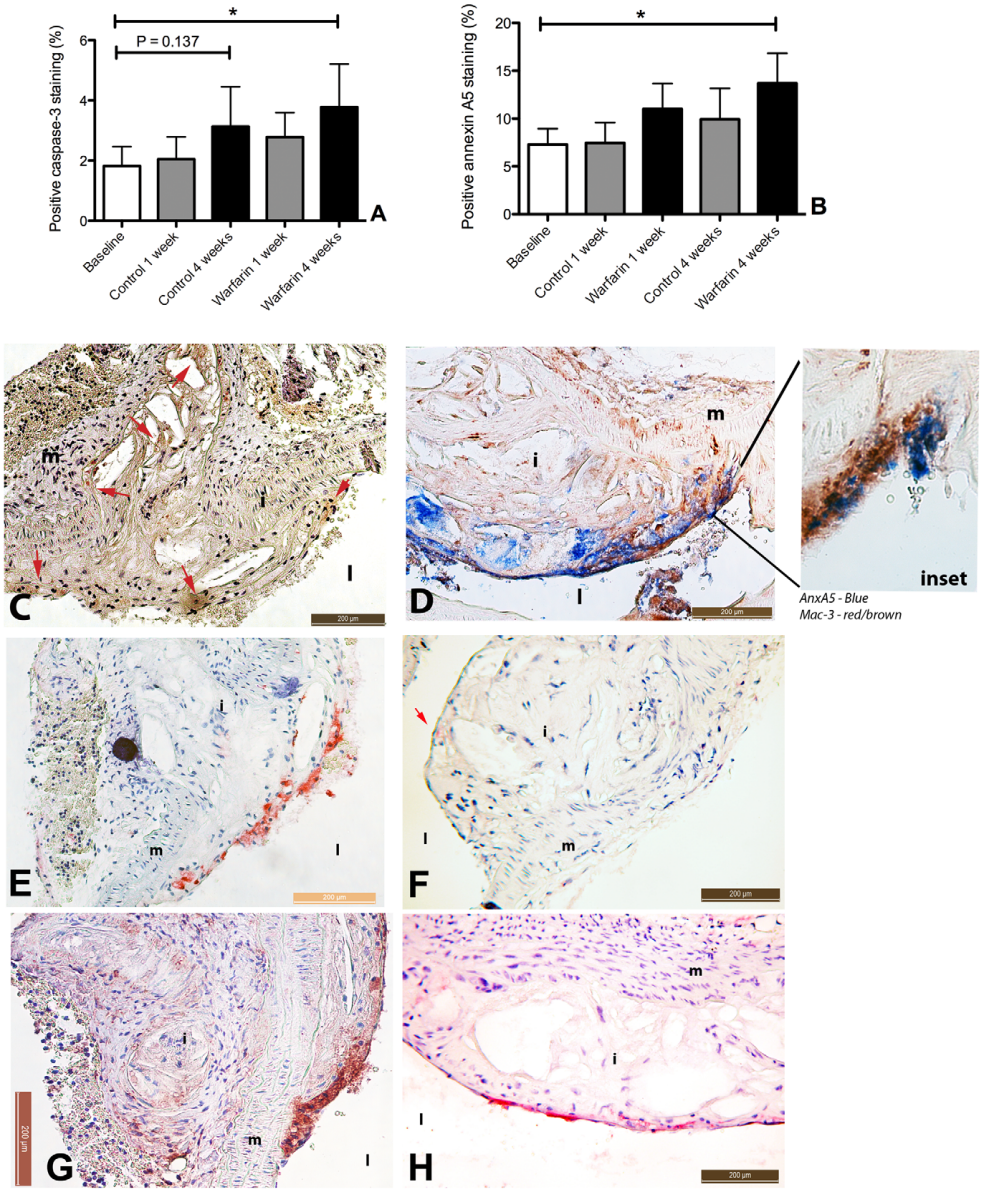
Warfarin treatment of apoE^−/−^ mice increases apoptosis in atherosclerotic plaque. Vascular calcification is closely linked to apoptosis. Therefore we stained sections for caspase-3. Moreover, all animals were injected with annexin A5-biotin, a protein with high affinity for phosphatidylserine (PS). Both cleaved caspase-3 (A,C) and annexin A5-biotin (B,D) increased in the warfarin treatment, with annexin A5 accumulation significantly increased after 4 weeks of warfarin treatment (B). Annexin A5 staining using AP vector blue confirmed PS externalization at different sites in the atherosclerotic plaques. Activated and apoptotic macrophages are known to express PS on their surface. We found co-localization of macrophages in the shoulder of the atherosclerotic plaque (Mac3; F) and PS (annexin A5 stained with AP vector red; E), indicating activated and apoptotic macrophages. In the inset annexin A5 (blue) localizes with Mac-3 staining (red/brown), indicating activated macrophages. Moreover, at baseline annexin A5 was only incidentally present at the surface of the plaque (G), whereas at the 4 weeks warfarin treatment, annexin A5 stained significantly positive (H). Statistically significant differences were determined by the Kruskal Wallis test. *P<0.05. i, intima; m, media; l, lumen.

Vascular calcification has been associated with apoptosis, both in media and intima [6], [24], [25], [26], [27]. Therefore we quantified apoptosis using two methods. Apoptosis was measured by caspase-3 staining (Figure 7A) and by annexin A5-biotin (Figure 7B), which was injected intravenously 30 minutes before sacrificing the mice. Warfarin caused a significant increase of apoptosis after 4 weeks of treatment. Similar to the calcium deposits, apoptotic cells were predominantly observed in plaques. Positive caspase-3 staining was noticed both in the plaque core and shoulder (Figure 7C). Likewise, annexin-A5-biotin positive staining was observed in the core, shoulders and the fibrous cap of the plaque (Figure 7D, inset and 7E,H) and co-localization with Mac3 revealed that part of the annexin A5 positivity was associated with macrophages, especially in the shoulder of the plaque (Figure 7D, inset, and 7F). At baseline, annexin A5-biotin staining was not significantly present (Figure 7G). We also measured the ratio of plaque macrophage/VSMC number but this ratio was not significantly different between the groups (data not shown).

**Figure 8:**
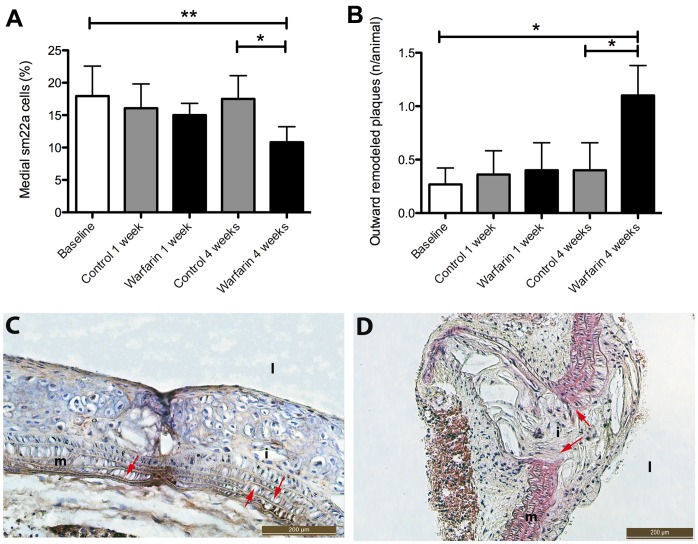
Warfarin induces medial VSMC loss and increases outward remodeling of atherosclerotic plaques, both indications of an instable plaque phenotype. An unexpected feature of warfarin treatment was the significantly increased number of outward remodeled plaques (B,D). Outward remodeling is, like plaque calcification, a feature of the instable rupture-prone atherosclerotic plaque. To explain this phenotype induced by warfarin we stained sections for the VSMC marker αSMactin. We found that warfarin treatment was associated with a significant loss of medial VSMCs (A,C). Statistically significant differences were determined by the Kruskal Wallis test. *P<0.05, **P<0.01. i, intima; m, media; l, lumen; o, outward remodeled plaque.

The intimal plaque vascular smooth muscle cells (VSMCs) are thought to be derived from the vascular media after having switched from a contractile into a synthetic phenotype. Therefore we measured VSMC number and elastin integrity in the medial layer underneath the atherosclerotic plaque. In warfarin treated animals, VSMCs were lower in number in the vascular media (Figure 8A,C), suggesting loss of elasticity and stability of the vascular media. Congruent with this finding, warfarin treated animals had significantly more outward remodeled plaques as demonstrated by disorganized tissue bulging outward through breaks of the elastic lamina (Figure 8B,D).

## Discussion

The present study demonstrates that VKA treatment is associated with accelerated calcification of atherosclerotic plaques in humans and is the first to demonstrate in a mouse model of atherosclerosis that VKA affects plaque phenotype negatively by enhancing features of plaque instability.

Vascular calcification precipitates in media of arteries along elastic fibers, also known as Mönckeberg’s sclerosis. Media calcification is closely linked with uremic risk factors. Calcification of intima is associated with atherosclerotic lesions. Calcium precipitates in close vicinity with lipid deposits and necrotic debris. It has been suggested that mechanisms of the two types of vascular calcification are distinct [28], [29]. A recent study reported a significant relationship between VKA treatment and coronary calcium score in atrial fibrillation patients without CAD [13]. This study did not address localization of calcium deposits in coronary arteries and, hence, could not provide insight into the arterial site affected by VKA treatment. Animal studies suggest that VKA treatment causes medial calcification similar to Mönckeberg’s sclerosis [23], [30], [31].

The present study investigated effects of VKA treatment on coronary calcium score in patients with suspected CAD, who underwent MDCT. Our study confirmed the relationship between VKA treatment and coronary calcification in this group of patients. Furthermore, MDCT offers the possibility to non-invasively assess lesions of the coronary arteries on morphology, degree of luminal stenosis and presence of calcium deposits [32]. Our data convincingly show that use and duration of VKA treatment correlate significantly with coronary plaque calcification. Although amount of coronary calcification was reported to have predictive value for cardiovascular events in subsets of patients [33], [34], the actual impact of calcification on plaque stability is controversial. In our patient population we did not find a clinical relevant difference in the occurrence of cardiovascular events (coronary revascularization, acute coronary syndrome, cardiovascular death) between VKA users and non VKA users. There are a few reasons contributing to the absence of a difference in cardiovascular events. First, we studied a small patient population. Secondly, the follow-up is relatively short (2.9±0.6 years) and it is not inconceivable that the follow-up period is too short to follow-up on cardiovascular event. Moreover, in case of the presence of coronary artery disease, patients were treated (life style changes, medication use) to prevent a cardiovascular event.

Recently it was postulated that increased risk for acute coronary events depends on size and location of calcium deposits. For instance, calcifications adjacent to or beneath the lipid necrotic core were assumed to be stabilizing [35]. On the other hand, elevated calcium scores were seen to be predictive of acute coronary events [36], [37] and culprit plaques often contain more but smaller calcium deposits (so-called spotty calcification) than stable plaques [20], [32], [38]. From biomechanical studies it was inferred that micro-calcifications in the fibrous cap have a destabilizing effect [21]. MDCT allows to map calcification to vascular anatomy and to distinguish calcified spots at millimeter resolution, insufficient to pick up micro-calcifications and to pinpoint localization. In order to reveal impact of VKA treatment on plaque stability we studied effects of warfarin on plaque calcification and phenotype in the apoE^−/−^ mouse model of atherosclerosis.

In this paper we demonstrate for the first time that warfarin increases plaque calcification in the apoE^−/−^ model. Warfarin-induced plaque calcification starts already after 1 week of administration, indicating that vitamin K-dependent mechanisms operate in developing plaques to suppress and limit pro-calcifying processes. cMGP is a well-known suppressor of vascular calcification [39], and a deficiency was previously shown to result in medial calcification [4], [5]. Transgenesis causing overexpression of MGP in apoE^−/−^ mice inhibited calcification of atherosclerotic lesions [18] demonstrating MGP’s modulating role in plaque calcification and indicating shared mechanisms by intimal and medial calcification. We observed a warfarin-induced downregulation of cMGP with concomitant upregulation of ucMGP in the plaque without affecting MGP-mRNA levels. Hence, we conclude that warfarin affects plaque calcification by inhibiting post-translational γ-carboxylation of MGP. This is in agreement with previous *in vitro* studies from our group demonstrating that warfarin treatment causes ucMGP production by VSMCs [10]. A recent study showed positive correlation between calcification of human coronary plaques and ucMGP expression in the plaque [40], strongly indicating that warfarin causes accelerated plaque calcification in human by a mechanism similar to that observed in the mouse model.

cMGP antagonizes BMP and is consequently linked to signaling networks regulating inflammation [41] and inducing VSMC differentiation [19] and apoptosis [42]. Thus, warfarin potentially affects plaque phenotype more profoundly than solely accelerating plaque calcification. We observed that warfarin treatment did not affect BMP-2 and -4 expression but increased collagen type-II and ALK expression concurring MGP regulated chrondrocytic trans-differentiation of VSMC [4], [43]. Furthermore, warfarin caused increased plaque apoptosis and loss of VSMC, both have been linked to calcification [25], [44] and progression towards unstable plaque [45], [46]. Thus, the loss of VSMCs underneath the plaque seen in our model may link to the observed increased number of outward remodeled plaques. In addition, it was recently shown that warfarin treated rats displayed increased MMP-9 activity in the vasculature which related to elastin degradation and vascular calcification [47]. Outward remodeled plaques have been linked to vulnerability of the plaque to rupture [48].

### Conclusions

We conclude that VKA ignite a cascade of responses leading to progressive calcification and destabilization of atherosclerotic plaques. Although the use of VKA thus may impart a risk factor for acute coronary events, the relatively safe historical profile of VKA suggests, however, otherwise. Detrimental effects of VKA could well be masked by their potent inhibitory effects on the coagulant system, an important determinant in atherothrombosis [49]. Nevertheless our findings support the growing need for alternative anticoagulants and underscore a need for anticoagulants that do not interfere with the vitamin K-cycle [50].

## Materials and Methods

### Study Population

Between January 2008 and April 2010, a total of 1,973 patients underwent a coronary calcium score scan as well as coronary computed tomographic angiography (CCTA) in our medical center. All patients were referred from the cardiology outpatient department because of cardiac symptoms suspected for CAD. CCTA was performed as part of the diagnostic work-up in these patients. Included were patients with complete data regarding their cardiac risk profile in order to calculate the Framingham Risk Score (FRS). The FRS is used to estimate the 10-year risk of cardiovascular disease and takes the following risk factors into account: age, gender, diabetes mellitus, smoking status, systolic blood pressure (treated as well as untreated), total cholesterol and high-density lipoprotein (HDL). Excluded were patients presenting to the emergency department because of acute chest pain. Patients with a known history of CAD, especially a history of myocardial infarction or coronary revascularization, were also excluded from this analysis. Patients were asked if they are using VKA. The duration of VKA use was verified in medical records. The VKA users (n = 133) were divided into tertiles, based on duration of VKA use (Table 1). Each VKA user was individually matched with a non-VKA user, based on equal FRS (Table 2). This study complies with the guidelines for good clinical practice and was performed in accordance with the Declaration of Helsinki.

### Risk Factor Assessment

Cardiac risk factors were prospectively collected by the referring cardiologists. Patients were classified as diabetics if they were treated with hypoglycemic medication or in case of a fasting plasma glucose ≥6.7 mmol/L (≥121 mg/dL). Patients were classified as smoker if they were current smoker. A positive family history was defined as having a first degree with a history of myocardial infarction or sudden cardiac death before the age of sixty. Total cholesterol, HDL, triglycerides and glucose were measured with the Synchron LX20 (Beckman Coulter, Brea, CA, USA). LDL was calculated using the Friedewald equation.

### CCTA Acquisition

Scans were performed using a 64-slice MDCT-scanner (Brilliance 64, Philips Healthcare, Best, the Netherlands) with a 64×0.625-mm slice collimation, a gantry rotation time of 420 ms and a tube voltage of 80 to 120 kV, depending on the patient’s height and weight. Patients received 50 mg Metoprolol tartrate orally, two hours before CCTA. When indicated, an additional dose of 5–20 mg Selokeen (AstraZeneca, Zoetermeer, the Netherlands) was given intravenously to lower the heart rate <65 beats per minute (bpm). All patients received 0,8 mg nitroglycerin spray (Pohl-Boskamp, Hohenlockstedt, Germany) sublingually just prior to CCTA to achieve optimal vasodilatation. Heart rate and ECG were monitored during the scan.

A non-enhanced scan was performed to determine coronary calcium score using the Agatston method [51]. Subsequently, CCTA was performed using 85–110 mL of contrast agent (Xenetix 350; Guerbet, Roissy CdG Cedex, France), which was injected in the antecubital vein at a flow rate of 6.0 mL/s, directly followed by 40 mL intravenous saline (6.0 mL/s) using a dual-head power injector. The most appropriate scan protocol was chosen based on the patient’s heart rate. In patients with a stable heart rate <65 bpm, a prospective ECG-gated ‘step and shoot’ protocol was used. In patients with a heart rate >65 bpm, a retrospective ECG-gated ‘helical’ protocol with dose modulation was used.

### Coronary Plaque Assessment

All scans were independently analyzed by two experienced imagers (IJ, WL), blinded for patient details, using source images in the Cardiac Comprehensive Analysis software (Philips Healthcare, Best, The Netherlands). In case of disagreement, consensus was reached by reviewing findings jointly.

The coronary calcium score was expressed as the Agatston score using dedicated calcium-scoring software (Philips Healthcare, Best, the Netherlands). All consecutive pixels (minimal total area of 1 mm^2^) with a threshold of 130 Hounsfield Units (HU) within the coronary arteries are considered as coronary calcification. The software automatically detects these coronary calcifications. The Agatston score for each calcification is calculated by multiplying the area of the lesion (mm^2^) by a weighting factor, which depends on the maximum density of the pixels. The total Agatston score is calculated by summing all individual calcifications [52]. The coronary artery tree was analyzed for the presence and severity of coronary artery disease, according to the 16-segment classification of the American Heart Association. Coronary plaques were defined as visible structures within or adjacent to the coronary artery lumen, which could be clearly distinguished from the vessel lumen and the surrounding pericardial tissue. Plaques were categorized as calcified (exclusively content with density >130 HU), non-calcified (exclusively content with density <130 HU) or mixed (characteristics of both calcified and non-calcified plaques). The degree of stenosis was classified as absent (no luminal stenosis), mild (<50% luminal stenosis), moderate (50–70% luminal stenosis) or severe (>70% luminal stenosis).

### Follow-up

Follow-up was performed in all patients regarding the occurrence of coronary revascularization procedures, cardiac mortality and acute coronary syndromes (ACS), including myocardial infarction and unstable angina requiring hospitalization. ACS was defined as typical angina pectoris, troponin T elevation (>0.01 μg/L) and ST-segment elevation/depression of ≥1 mm, or at least two of these characteristics together with invasive angiographic confirmation of a culprit lesion [53]. Patients were seen by their cardiologist on a regular basis, and all hospital visits, both outpatient department visits as well as emergency room visits, were recorded in the electronic patient records. Additionally, the national mortality records were checked.

### Animals and Diet

Both male and female apoE^−/−^ mice were purchased from the Maastricht University. Mice were aged 10 weeks when entering the study and all animals were housed in normal cages with free access to water and the provided foods. Irradiated (0.9Mrad) vitamin K-deficient WTD (0.25% cholesterol and 15% cocoa butter) was from Arie Blok, Woerden, the Netherlands. Vitamin K_1,_ dissolved in corn oil, was added to the vitamin K-deficient food in the required amounts. The VKA warfarin was added directly to the food. The Experimental Animal Experimental Committee of the Maastricht University approved all described animal protocols.

The VKA/vitamin K_1_ model is based on high doses of VKA administered to mice with concomitant administration of vitamin K_1_ to overcome the antagonism of VKA in liver but not in extrahepatic tissues such as the vasculature [23]. Thus the effects of VKA on extrahepatic tissues can be studied without the animals suffering from major bleedings. To induce atherosclerosis, mice (n = 40) received a WTD containing vitamin K_1_ (1.5 mg/g food) for three months. After 12 weeks of treatment, 8 mice were sacrificed to monitor baseline atherosclerosis (t = 0; baseline group). The remaining mice were divided into two groups of 16 mice. The first group continued the WTD + vitamin K_1_ (1.5 mg/g food) (VK_1_ group), the second group received the WTD + vitamin K_1_ (1.5 mg/g food) + warfarin (3.0 mg/g food) (VK_1_&W group). These two diets were continued for another week and 8 mice from each group were sacrificed. The remaining mice continued the VK_1_ and VK_1_&W diet and were sacrificed 4 weeks after the start of the experimental diets.

### Experimental Animal Procedures

Thirty minutes prior to sacrificing the mice, annexin A5-biotin [54] was injected via the tail vein (16 μg/gram body weight). After 30 minutes, mice were anesthetized with 4% isoflurane and kept anesthetized using isoflurane 1.5 - 2.5%. Blood was collected in 105 mM trisodium citrate via the portal vein and plasma aliquots were frozen at −80°C. Before collecting all required tissues, the vasculature was perfused with a sterile vasodilating saline solution (150 mM saline, 2.5 mM CaCl_2_, and 100 pM sodium nitroprusside in HEPES, pH 7.3) via the portal vein. The aortic arch, thoracic and abdominal aorta (bifurcation till one cm above), and right and left carotid artery were dissected, transferred to a physiological salt solution in a silicon-coated Petri dish, and adipose and connective tissue were carefully removed. The abdominal aorta was frozen in liquid nitrogen for assessment of the calcium content. The aortic arch and right carotid artery were fixed in 4% (v/v) HEPES buffered formaldehyde containing 2.5 mM calcium and 150 mM saline and transferred after one hour to 1% (v/v) HEPES-buffered formaldehyde containing 2.5 mM calcium and 150 mM saline and kept at 4°C for 24 hours. Vascular tissue was next transferred to 70% ethanol before use for embedding and subsequent immunohistochemistry. The thoracic aorta was snap frozen in liquid nitrogen and stored at −80°C for alkaline phosphatase (ALK) analysis. The left carotid artery was snap frozen in liquid nitrogen and stored at −80°C for mRNA analysis.

### Analysis of Vascular Lesions in ApoE^−/−^ Mice

Cryosections and paraffin sections, 4 µm thick, were cut from the abdominal aorta starting (bifurcation of iliaca till one cm higher) and aortic arch. Cryosections were stained with hematoxylin/eosin (HE) for histochemical analysis. Masson’s trichrome stain and von Kossa stain were performed. Quantitative analysis of lesions was performed using ImageJ histomorphometry software on at least 4 sections from each mouse.

### Antibodies and Chemicals

Parallel sections were stained with monoclonal rat anti- mouse Mac3 (1∶30, BD Pharmingen, #S2031-30), aSMactin (1∶200, Abcam, #AB-15734), BMP2 (1∶50, Santa Cruz, #sc-6895), cleaved caspase-3 (1∶100, Cell signaling tech, #9661S), Collagen type2 (1∶100, Developmental Studies Hybridoma Bank, #II-II6B3), MGP (against various epitopes, Vascular Products BV, Maastricht, the Netherlands), designated as anti-cMGP (1∶250, recognizing carboxylated MGP; cMGP), and anti-ucMGP (1∶250, recognizing uncarboxylated MGP; ucMGP), respectively.

Biotinylation of mono- and polyclonal antibodies, and subsequent streptavidin-HRP labeling were performed using the Dako-Kit System-HRP (LSAB2, #K0673). Antibodies were visualized with a Nova-RED substrate (Vector #SK-4800, Vector Laboratories, Inc). In case of annexin A5-biotin, staining was performed using streptavidin-AP. Visualization was done using AP-vector red or AP-vector blue. Sections were counterstained with hematoxylin (Klinipath, #4085-9002) and mounted with imsol (Klinipath, #7961) and entellan (Merck #7961). In negative controls, incubation with primary antibody was omitted.

Vitamin K_1_ and warfarin were purchased from Sigma (Saint Louis, USA). All chemicals were of analytical grade or better.

### Biochemical and Immunohistochemical Measurements of apoE^−/−^ Tissues

Tissue calcium was determined after lyophilization and expressed per mg dry weight; the freeze-dried tissues were extracted with a tenfold excess (v/w) of 10% formic acid (overnight at 4°C) and calcium concentrations were measured using atomic absorption-spectrometry (AAS, Department of Clinical Chemistry, University Hospital Maastricht, The Netherlands). For the ALK assay, part of the vessel was homogenized in ice-cold TBS supplemented with protein inhibitor cocktail using a Polytron-type homogenizer. ALK activity was measured colorimetrically at 405 nm using a p-nitrophenyl phosphate substrate (Sigma). The total ALK level per vessel part was normalized to the protein content for comparisons. Immunohistochemistry was performed after embedding the vascular tissues in paraffin and subsequent sectioning (4 µm thick). Each seventh section was used for calcium detection by von Kossa staining. Each antibody staining was performed in one batch. Annexin A5-biotin was stained using streptavidin-AP, after blocking for endogenous peroxidase. Appropriate negative controls were used for antibody and annexin A5 staining. The relative extent of staining was measured using a microscope coupled to a computerized morphometry system (quantimed 570, Leica, the Netherlands). Quantification was expressed as area in mm^2^ or as percentage staining of the total plaque area. To reliably compare staining of different antibodies both microscope and camera adjustments were kept constant [6], [55].

Calcium, phosphate, cholesterol, and triglycerides were measured in plasma. Lipids were measured at the end of the experiments by standard enzymatic techniques.

### RNA Isolation and Quantitative Reverse Transcription Real-time PCR

Messenger RNA was extracted using RNeasy Mini Kit for kidney and lung tissues and RNeasy Fibrous Tissue Kit with proteinase K digestion (Qiagen, Hilden, Germany) before RNA extraction for aorta to maximize mRNA yield. mRNA concentration and 260 nm/280 nm ratio were measured by Nanodrop 1000 (Thermo Scientific, USA). Integrity and amount of mRNA were analyzed by capillary electrophoresis (Agilent Bioanalyzer 2100; Agilent Technologies, Boblingen, Germany). Reverse transcription and real-time PCR were performed using the TaqMan Gene Expression Master Mix (PE, Applied Biosystems, Foster City, CA, USA) and the 7500 Fast Real Time PCR System according to the manufacturer’s instructions. The following temperature profile was used: 2 min at 50°C, 10 min at 95°C, and 40 cycles at 95°C following 60°C.

All primers (TaqMan Gene Expression Assays) were from Applied Biosystems, including MGP (Mm00485009_m1), SM22-α (Mm00441660_m1), RunX2 (Mm00501578_m1), VEGF (Mm01281449_m1), BMP-2 (Mm01340178_m1), BMP-4 (Mm00432087_m1), MMP-2 (Mm00485009_m1) and MMP-9 (Mm00100985_m1). Glyceraldehyde-3-phosphate dehydrogenase (GAPDH) was used as housekeeping target (Mm03302249_g1). The expression of each target gene was normalized to GAPDH. For calculating relative expression levels the ΔCt (cycle threshold) method was used.

### Statistical Analysis

Statistical analyses were performed using SPSS software (version 17.0, SPSS Inc., Chicago, IL, USA). Categorical baseline characteristics are expressed as numbers (percentages) while continuous variables are expressed as means ± SD. To test differences between patients in the tertiles for statistical significance, we used the Pearson chi-square test for categorical variables and analysis of variance (ANOVA) for continuous variables. Values in animals are expressed as mean ± SD. The difference between two groups was determined by Kruskal Wallis test. Differences for multiple comparisons were determined by ANOVA with Bonferroni correction. Differences were considered to be significant at p<0.05.
